# Clinical features and prognostic factors of children with profound sudden sensorineural hearing loss

**DOI:** 10.3389/fped.2022.1023781

**Published:** 2022-11-07

**Authors:** Ling Xiao, Shuping Su, Jia Liang, Ying Jiang, Yan Shu, Hongbing Yao, Ling Ding

**Affiliations:** ^1^ Department of Otolaryngology-Head and Neck Surgery, Children's Hospital of Chongqing Medical University, Chongqing, China; ^2^ National Clinical Research Center for Child Health and Disorders, Chongqing, China; ^3^ Ministry of Education Key Laboratory of Child Development and Disorders, Chongqing, China; ^4^ Chongqing Key Laboratory of Pediatrics, Chongqing, China

**Keywords:** sudden sensorineural hearing loss (SSNHL), profound, prognosis, children, intratympanic steroid

## Abstract

**Objective:**

To investigate the clinical features and factors affecting the prognosis of children with profound sudden sensorineural hearing loss (SSNHL).

**Methods:**

We retrospectively analyzed the clinical data of 147 children with profound SSNHL who received inpatient treatment at our department from January 2016 to January 2021. All children were administered with systemic steroid therapy and/or intratympanic steroid (ITS) treatment for 2 weeks. Statistical analyses were performed for the clinical features, treatment effectiveness, and factors affecting the prognosis using SPSS 23.0.

**Results:**

The median age of the study population was 8 (6–10) years. The median treatment onset time was 8 (4–20) days. The most common concomitant symptom was tinnitus (45.58%). Laboratory findings showed that the percentages of children with abnormal leukocytes was 25.85%, abnormal platelet counts was 17.01%, abnormal cytomegalovirus IgG antibodies was 36.73% and abnormal Epstein–Barr (EB) virus IgG antibodies was 41.50%. The overall recovery rate of the treatment was 20.04%. The univariate analysis showed that age, treatment onset time, tinnitus, and ITS treatment were associated with the prognosis (*p* < 0.05). Regarding laboratory findings, the neutrophil count, lymphocyte count, and neutrophil-to-lymphocyte ratio differed significantly between the effective and invalid treatment effect groups (*p* < 0.05). The multivariable logistic regression analysis showed that treatment onset time [odds ratio (OR) = 0.936, 95% confidence interval (CI): 0.881–0.994] and ITS treatment (OR = 0.174, 95% CI: 0.044–0.0687) correlated with hearing recovery (*p* < 0.05).

**Conclusion:**

In this study, the earlier the treatment start time of children with profound SSNHL, the better was the prognosis. Further, ITS could be an effective treatment option.

## Introduction

Sudden sensorineural hearing loss (SSNHL) is defined as a rapidly developed hearing loss (occurs within a 72-h window) with an increased pure-tone threshold over 30 dB affecting at least three consecutive frequencies (1). Four types of audiogram configurations were defined based on the hearing loss pattern: ascending, descending, flat, and profound (2). The profound audiogram refers to the presence of a similar threshold across the frequency range and a hearing threshold over 80 dB HL (2, 3). Profound SSNHL accounting for approximately 34.1% of all cases of SSNHL, is associated with severe hearing loss and a poor prognosis, with 29.8% of the patients achieving a certain degree of recovery and only 3.6% of the patients achieving full recovery (4).

The incidence of SSNHL in children (age <18 years) is low, with only 20–30 cases per 100,000 children per year (5). Only 6.6%, 3.5%, and 1.2% of the patients with SSNHL are aged under 18 (6), 14 (7), and 9 years (8), respectively. In children, 55.3% of the cases of SSNHL are of profound hearing loss, and its effective treatment rate was found to be 31% (9). The profound SSNHL is the most severe form of hearing loss. Without timely treatment, it leads to permanent hearing loss, seriously affecting children's language and cognitive developments and increasing the burden on family and society.

The etiology and pathogenesis of SSNHL in children are unclear. Further, the treatment outcomes and factors affecting the prognosis are unknown, with no guidelines for the diagnosis or treatment. Most previous studies analyzed the risk factors and found a correlation between the hearing curve and the treatment effect (2, 10). However, factors affecting the prognosis of different types of SSNHL in children have not been investigated. Therefore, the aim of the present study was to retrospectively analyze the clinical characteristics and factors affecting the prognosis of profound SSNHL in children.

## Materials and methods

### Clinical data

We retrospectively enrolled 147 children diagnosed with profound SSNHL between January 2016 and January 2021 from the Children's Affiliated Hospital of Chongqing Medical University. We collected the clinical data, including the age, sex, affected side, treatment onset time, initial hearing level, accompanied tinnitus, vertigo, and aural fullness, hearing levels before and after treatment, complete blood count, serum biochemical findings, coagulation function, and viral serology results. The requirement to obtain informed consent was waived owing to the retrospective nature of the study. However, written informed consent was obtained from the guardians of the children for administering systemic steroid therapy (SST) and intratympanic steroid (ITS) treatment. The study protocol was approved by the Medical Ethics Committee of the Children's Affiliated Hospital of Chongqing Medical University.

All children met the diagnostic criteria for SSNHL in children laid down by the American Academy of Otolaryngology-Head and Neck Surgery (AAO-HNS) (1). Inclusion criteria were: SSNHL diagnosed based on sudden, unexplained hearing loss within 72 h of more than 30 dB HL for at least three adjacent frequencies; age of 3–18 years; profound SSNHL determined based on full-frequency hearing loss with an average hearing level >80 dB HL; and availability of complete audiological examination and experimental results. Exclusion criteria were: hearing loss due to auditory nerve or middle or outer ear disease based on acoustic impedance, otoacoustic emissions, auditory brainstem response (ABR), otoscopy, ear computed tomography, or ear magnetic resonance imaging findings; suspected hearing loss caused by noise, medication, trauma, neurological disorders, or genetic factors; comorbidities; and data unavailability.

### Inspection method

#### Laboratory examination

Routine blood, serum biochemical, coagulation function, and viral serology tests were performed. We collected the white blood cell count, platelet count, neutrophil count, lymphocyte count, neutrophil-to-lymphocyte ratio (NLR), platelet-to-lymphocyte ratio (PLR), triglyceride level, cholesterol level, D2 polymer level, fibrinogen level, and cytomegalovirus and Epstein–Barr (EB) virus IgG and IgM antibodies.

#### Hearing tests

We performed hearing tests, including pure-tone audiometry, ABR, auditory steady state response (ASSR), and distortion product otoacoustic emission. Most children underwent pure-tone audiometry (according to ISO8253-1 standards), but those who could not cooperate in the pure-tone audiometry procedure underwent combined ABR and ASSR.

#### Therapeutic method

All children were treated for 2 weeks after admission. The primary treatment was SST with 1 mg/kg/day intravenous methylprednisolone for 5 days, with the maximum dose not exceeding 40 mg, followed by dose tapering. Twenty-seven children were administered with ITS once every 2 days for a total of 5 times using otoendoscopy. The ITS treatment was performed as follows: The child was placed in a seated position with the head tilted to the unaffected side; after local anesthesia with lidocaine, a tympanic membrane needle was introduced into the anterior portion of the tympanic membrane; subsequently, 0.4–0.8 ml of 4 mg/ml methylprednisolone was instilled in the middle ear cavity; the child was instructed to lie down and avoid swallowing for 15 min. Children on oral hormones were provided outpatient treatment and excluded from this study. After 2 weeks of treatment, hearing tests were performed again. Hearing levels were compared before and after the treatment to evaluate the treatment efficacy.

### Treatment efficacy determination

Audiometry was performed at the initial visit and after 2 weeks of treatment. According to the Chinese Medical Association of Otolaryngology criteria for the sudden deafness treatment effect (3), complete recovery is defined as improvement in final hearing to a normal or pretreatment level; partial recovery is defined as a hearing improvement of more than 30 dB HL; slight recovery is defined as a hearing improvement of 15–30 dB HL; and no recovery is defined as a hearing improvement of less than 15 dB HL. The overall recovery rate was calculated with the following formula: (complete recovery + partial recovery + slight recovery)/total no. of cases × 100%.

### Statistical analysis

Data were analyzed using SPSS 23.0 statistical software (IBM, Armonk, NY, USA). Normally distributed continuous data are expressed as mean ± standard deviation. Non-normally distributed data are expressed as median (1/4 quantile–3/4 quartile). Classification data are expressed as frequency (percentage). The *t*-test was performed for two-group comparisons of normally distributed data and the Mann–Whitney *U* test for non-normally distributed data. Associated factors were analyzed using the *χ*^2^ or rank test. A univariate analysis was performed for the relationship of hearing recovery with the sex, age, affected side, treatment onset time, initial hearing level, accompanied vertigo, tinnitus, and ear tightness, and blood parameter findings. A multivariate logistic regression analysis was performed for variables that were statistically significant in the univariate analysis to explore their impact on the prognosis. Statistical significance was set at *p* < 0.05.

## Results

### Clinical features of profound SSNHL in children

We enrolled 147 children, including 72 (48.98%) boys and 75 (51.02%) girls, aged 8 (6–10) years. The right and left ears were affected in 66 (44.90%) and 81 (55.10%) children, respectively. The treatment onset time was 8 (4–20) days. The initial hearing level was 103 (95–113) dB. Tinnitus, vertigo, ear fullness, and upper respiratory tract infection were found in 67 (45.58%), 26 (17.69%), 24 (16.33%), and 44 (29.93%) children, respectively (Table 1).

**Table 1:** Clinical features of children with profound sudden sensorineural hearing loss (*n* = 147).

Characteristics	
Sex (male:female)	72:75
Age (years)	8 (6, 10)
Side (left:right)	66:81
Treatment onset time (days)	8 (4–20)
Initial hearing level (dB)	103 (95–113)
Post-treatment hearing level (dB)	62 (37–95)
Tinnitus	67 (45.58%)
Vertigo	26 (17.69%)
Ear fullness	24 (16.33%)
Upper respiratory tract infection	44 (29.93%)

### Laboratory examination results

Routine blood tests showed abnormal leukocyte, platelet, neutrophil, and lymphocyte counts in 38 (25.85%), 25 (17.01%), 21 (14.29%), and 17 (11.56%) children, respectively. Biochemical tests showed abnormal cholesterol and triglyceride levels in four (2.72%) and six (4.08%) children, respectively. Coagulation tests showed abnormal D2 polymer and fibrinogen levels in three (2.04%) children. Cytomegalovirus IgM and IgG antibodies were found in four (2.72%) and 54 (36.73%) children, respectively, while EB virus antibodies were found in five (3.40%) and 61 (41.50%) children, respectively (Table 2).

**Table 2:** Laboratory examination results of children with profound sudden sensorineural hearing loss.

		Normal	Abnormal
Routine blood test	WBC count	109 (74.15%)	38 (25.85%)
	Platelet count	122 (82.99%)	25 (17.01%)
	Neutrophil count	126 (85.71%)	21 (14.29%)
	Lymphocyte count	130 (88.44%)	17 (11.56%)
Serum biochemical	Cholesterol level	143 (97.28%)	4 (2.72%)
	Triglyceride level	141 (95.92%)	6 (4.08%)
Coagulation test	D2 polymer level	144 (97.96%)	3 (2.04%)
	Fibrinogen level	144 (97.96%)	3 (2.04%)
Antiviral antibody test	Cytomegalovirus IgM	143 (97.28%)	4 (2.72%)
	Cytomegalovirus IgG	93 (63.27%)	54 (36.73%)
	EB virus IgM	142 (96.60%)	5 (3.40%)
	EB virus IgG	86 (58.50%)	61 (41.50%)

WBC, white blood cell; EB, Epstein–Barr.

### Univariate analysis of the prognosis of children with profound SSNHL

Complete recovery, partial recovery, slight recovery, and no recovery were found in one (0.07%), nine (6.1%), 20 (13.6%), and 117 (79.6%) children, respectively, with a total response rate of 20.4%. SST alone was performed in 120 children and was effective in 17 (14.17%) children, while SST combined with ITS administration was performed in 27 children and was effective in 13 (48.15%) children. The univariate analysis showed that the age, treatment onset time, tinnitus, and ITS treatment were associated with the prognosis (*p* < 0.05), while the sex, affected side, initial hearing level, or accompanied vertigo, ear fullness, or upper respiratory infection was not associated with the treatment effect (*p* > 0.05; Table 3). The neutrophil count, lymphocyte count, and NLR significantly differed between the effective and invalid treatment effect groups (*p* < 0.05), while the leukocyte count, platelet count, PLR, triglyceride level, cholesterol level, D2 polymer level, fibrinogen level, or antiviral antibody positivity rate did not differ between the two groups (*p* > 0.05; Table 4).

**Table 3:** Univariate analysis of the prognosis of children with profound sudden sensorineural hearing loss.

Variables			*n*	Recovery (*n* = 30)	No recovery (*n* = 117)	*χ*^2^/*z*-value	*p*-value
Sex	Male		72	15 (20.83%)	57 (79.17%)	0.16	0.900^a^
	Female		75	15 (20%)	60 (80%)		
Affected side	Left		66	12 (18.18%)	54 (81.82%)	0.366	0.545^a^
	Right		81	18 (22.22%)	63 (77.78%)		
Age (years)			147	8 (11–12)	7 (6–9)	3.862	0.000^b^^,^*
Treatment onset time (days)			147	4 (1.75–7)	10 (5–30)	4.018	0.000^b^^,^*
Initial hearing level (dB)			147	99 (87.75–108)	106 (95–113)	1.191	0.055^b^
Accompanying conditions	Tinnitus	Yes	67	20 (29.85%)	47 (70.15%)	6.758	0.009^a^^,^*
		No	80	10 (12.50%)	70 (87.50%)		
	Vertigo	Yes	26	8 (30.77%)	18 (69.23%)	2.088	0.149^a^
		No	121	22 (18.18%)	99 (81.82%)		
	Ear fullness	Yes	24	7 (29.17%)	17 (70.83%)	1.155	0.244^a^
		No	123	23 (18.70%)	100 (81.30%)		
Upper respiratory tract infection	Yes		44	12 (27.27%)	32 (72.73%)	1.822	0.177^a^
	No		103	18 (17.48%)	85 (82.52%)		
ITS	Yes		27	13 (48.15%)	14 (51.85%)	15.669	0.000^a^^,^*
	No		120	17 (14.17%)	103 (85.83%)		

ITS, intratympanic steroid.

^a^ Pearson's chi-square test.

^b^ Mann–Whitney *U*-test.

* *p* < 0.05.

**Table 4:** Comparison of laboratory examination results between patients with and without hearing recovery.

Variables		Recovery	No recovery	*t*-value	*p*-value
Routine blood test	WBC count (10^9^/L)	8.97 ± 2.04	9.32 ± 2.64	0.546	0.585^a^
	Platelet count (10^9^/L)	275.33 ± 84.63	290.27 ± 75.21	0.673	0.501^a^
	Neutrophil count (10^9^/L)	5.26 ± 1.97	6.67 ± 2.69	2.610	0.009^a^^,^*
	Lymphocyte count (10^9^/L)	2.68 ± 0.90	2.32 ± 0.75	2.185	0.029^a^^,^*
	NLR	2.29 ± 1.50	3.20 ± 1.70	3.216	0.001^a^^,^*
	PLR	119.79 ± 69.25	138.63 ± 61.48	1.762	0.078^a^
Serum biochemical test	Cholesterol level (mmol/L)	4.28 ± 0.71	4.19 ± 0.60	0.680	1.827^a^
	Triglyceride level (mmol/L)	1.20 ± 0.65	0.94 ± 0.45	0.496	0.068^a^
Coagulation test	D2 polymer level (mg/L)	0.35 ± 0.66	0.21 ± 0.24	0.074	0.941^a^
	Fibrinogen level (g/L)	2.27 ± 0.73	2.24 ± 0.45	1.072	0.284^a^
Antiviral antibody test	Cytomegalovirus IgM (+)	1	3	0.053	0.818^c^
	Cytomegalovirus IgG (+)	14	40	1.600	0.206^b^
	EB virus IgM (+)	1	4	0.001	0.982^c^
	EB virus IgG (+)	16	45	2.175	0.140^b^

WBC, white blood cell; NLR, neutrophil-to-lymphocyte ratio; PLR, platelet-to-lymphocyte ratio; EB, Epstein–Barr.

^a^ *t*-value.

^b^ Pearson's chi-square test.

^c^ Fisher's exact test.

* *p* < 0.05.

### Multivariate analysis of the treatment efficacy of children with profound SSNHL

The multivariate logistic regression analysis showed that the treatment onset time (odds ratio = 0.936, 95% confidence interval: 0.881–0.994) and ITS treatment (odds ratio = 0.174, 95% confidence interval: 0.044–0.0687) were associated with treatment efficacy (*p* < 0.05; Table 5).

**Table 5:** Logistic multivariate regression analysis of the treatment efficacy in children with profound sudden sensorineural hearing loss.

Variates	*B*	SE	Wald	*p*-value	OR	95% CI
Age	0.224	0.115	3.766	0.052	1.251	0.998–1.568
Treatment onset time	−0.066	0.031	4.716	0.030*	0.936	0.881–0.994
Tinnitus	−0.278	0.536	0.269	0.604	0.757	0.265–2.166
ITS	−1.746	0.699	6.235	0.013*	0.174	0.044–0.687
Neutrophil count	−0.248	0.202	1.511	0.219	0.780	0.526–1.159
Lymphocyte count	0.572	0.508	1.271	0.260	1.772	0.655–4.793
NLR	−0.118	0.413	0.082	0.775	0.889	0.395–1.998

ITS, intratympanic steroid; NLR, neutrophil-to-lymphocyte ratio; SE, standard error; OR, odds ratio; CI, confidence interval.

* *p* < 0.05.

## Discussion

In this study, we analyzed the clinical characteristics of children with profound SSNHL and focused on investigating the risk factors to help evaluate the treatment effect. The age of onset of profound SSNHL was 8 years with no sex predilection, consistent with previous studies (11, 12). All children had unilateral SSNHL with no significant difference between the left and right sides (13). A frequent comorbidity with SSNHL was tinnitus, accounting for 45.58% of the cases, consistent with previous studies (14). Abnormal leukocyte count was found in 25.85% of the children. Cytomegalovirus IgM and IgG antibodies were found in 2.72% and 36.73% of the children, and EB virus IgM and IgG antibodies were found in 3.40% and 41.50% of the children, respectively, indicating that viral infection may be a pathogenic factor of profound SSNHL in children, similar to that reported by Pitaro et al. (14). In this study, 25 (17.01%) patients had elevated platelet count, and three (2.04%) patients had abnormal coagulation function, indicating that platelet count elevation and abnormal coagulation function may be pathogenic factors of profound SSNHL in children as they promote thrombosis and lead to cochlear microcirculation disorder. Viral infection and microcirculation disorder in children with SSNHL with the profound audiogram pattern can lead to thrombosis and vascular damage.

The effective treatment rate for profound SSNHL was 20.4% in the present study; it is associated with a poor prognosis and many influencing prognostic factors. In this study, age was associated with the treatment effectiveness of profound SSNHL in children. Patients were older (11–12 years) in the effective treatment group than in the invalid treatment effect group (6–9 years). This may be because younger children have poor perception and expression abilities, hindering the timely detection of unilateral deafness by parents, unlike older children. Kim et al. compared the prognosis of SSNHL between children (4–12 years) and adolescents (>12 years) and found that children had a significantly worse prognosis compared to adolescents (15), similar to our study findings. However, neither Qian et al. nor Li et al. found any correlation between age and SSNHL prognosis (2, 11).

In this study, children with tinnitus (29.85%) had a significantly better prognosis than those without tinnitus (12.50%; *p* < 0.05), indicating that tinnitus was a positive prognostic factor, consistent with previous studies (2, 6, 15). The presence of tinnitus suggests that inner ear hair cells are functional. Kruntorád et al. found that children with tinnitus have a 54.5% chance of hearing restitution, while those without tinnitus have a 35.3% chance (16). The recovery rate of combined tinnitus was higher than that reported in our study. A meta-analysis also revealed that patients with tinnitus had 2.2 times better hearing with partial or complete improvement than those without tinnitus (10). However, Li et al. analyzed prognostic factors of SSNHL in 101 children (113 ears) and found that tinnitus was not related to the prognosis (17). Ashtiani et al. found that tinnitus is a negative prognostic factor (18). The role of tinnitus in the prognosis of SSNHL with the profound audiogram pattern is controversial and should be further explored. In addition, vertigo and ear fullness were not associated with the prognosis (*p* > 0.05), consistent with previous studies (11, 16, 17).

A meta-analysis classified prognostic biomarkers of SSNHL in adults into six categories: inflammation, metabolism, coagulation, immunity, oxidation, and others. The analysis of the relationship of these factors with the prognosis of SSNHL revealed that low monocyte count, NLR, and fibrinogen level were correlated with the prognosis (19). The meta-analysis conducted by Cao et al. showed that the NLR, PLR, neutrophil count, and lymphocyte count were closely associated with the prognosis of SSNHL in adults (20). Studies also identified that NLR was a prognostic biomarker of SSNHL in adults (21, 22). Ha et al. showed that NLR had some prognostic value for SSNHL in children (23). In this study, blood routine, serum biochemical, coagulation, and viral antibody tests revealed that the neutrophil count and NLR were lower while the lymphocyte count was higher in the effective treatment group than in the invalid treatment effect group, consistent with Lee et al.'s study (24). Elevated neutrophil count and NLR and reduced lymphocyte count were associated with a poor prognosis, possibly because elevated NLR increases inflammation, and elevated neutrophil count can cause endothelial damage, leading to microcirculation damage, in addition to lymphocyte apoptosis occurrence during inflammation, reducing the lymphocyte count. The multivariate analysis showed that none of these parameters were independent risk factors for the prognosis of profound SSNHL in children, suggesting that these factors do not fully determine the onset or prognosis of profound SSNHL in children. Further basic and clinical studies should be performed in the future.

The AAO-HNS guidelines recommend early initiation of treatment, i.e., within 2 weeks of disease onset, for SSNHL (1). In this study, children with effective treatment had visited much earlier than those with invalid treatment effect, with an average treatment onset time of approximately 8 days. Liu et al. found that in adolescents with sudden deafness, early treatment (usually <8 days) had a better prognosis (25). Bulgurcu et al. also found that prognostic factors had no significant effect when the treatment for idiopathic sudden hearing loss was initiated within 7 days from the onset of hearing loss (26). Chung et al. performed a multivariate analysis of the prognostic factors of SSNHL and found that early treatment was associated with a good prognosis (6). Our study found that treatment onset time was an independent risk factor affecting the prognosis of SSNHL with the profound audiogram pattern, consistent with previous studies (2, 9, 27). This may be related to the microcirculation disorder of the inner ear. The later the treatment begins, the more likely it is to cause hypoxia and hair cell damage in the inner ear. Inner ear hair cell damage is reversible when treated early but may be permanent and irreversible otherwise. SSNHL in children is a medical emergency in the department of otolaryngology and should be diagnosed and treated as early as possible for good treatment efficacy.

The guidelines also recommended SST to treat SSNHL with ITS administration as a salvage treatment within 2–6 weeks (1). SST has become the most widely accepted treatment for both adults and children. However, studies investigating ITS administration in children have been scarce. ITS administration requires patient cooperation, and young children are less cooperative. It also carries risks of pain, bleeding, and infection, limiting its application in children (28). Previous studies revealed that ITS administration could benefit children (15). In this study, 27 children were administered with SST + ITS, with a significantly higher treatment response rate (48.15%) than those who were administered with SST alone (14.17%; *p* < 0.05), which suggests that ITS could be effective for profound SSNHL in children. SST is the first-line treatment of SSNHL in children, and salvage therapy with ITS treatment is helpful after treatment failure with SST (2, 29). However, data are lacking on ITS administration in children with profound SSNHL. Therefore, further large-scale multicenter prospective studies are required to investigate the efficacy and safety of ITS administration in children.

This study had some limitations. First, it was a retrospective study with a small sample size, which may lead to bias. Second, the treatment effect analysis of SSNHL were performed according to the Chinese standards, which differ from the international and Siegel standards. Third, ITS treatment in childen is controversial. This was not a randomized controlled trial. Future large-scale prospective studies should be performed with international standards for the diagnosis and treatment of SSNHL in children.

## Conclusion

In this study, profound SSNHL in children had a poor prognosis. The prognosis was associated with the treatment onset time. Therefore, it should be treated as early as possible to improve treatment efficacy and restore hearing in children to avoid permanent hearing loss. ITS administration could be effective for profound SSNHL in children.

## Data Availability

The original contributions presented in the study are included in the article/Supplementary Material, further inquiries can be directed to the corresponding author/s.
